# Rehabilitation assisted by Space technology—A SAHC approach in immobilized patients—A case of stroke

**DOI:** 10.3389/fphys.2022.1024389

**Published:** 2023-01-18

**Authors:** Chrysoula Kourtidou-Papadeli, Christos Frantzidis, Ilias Machairas, Christos Giantsios, Emmanouil Dermitzakis, Nikolaos Kantouris, Evdokimos Konstantinids, Panagiotis Bamidis, Joan Vernikos

**Affiliations:** ^1^ Laboratory of Medical Physics, Biomedical Engineering & Aerospace Neuroscience (BEAN), School of Medicine, Faculty of Health Sciences, Aristotle University of Thessaloniki, Thessaloniki, Greece; ^2^ Greek Aerospace Medical Association and Space Research (GASMA-SR), Thessaloniki, Greece; ^3^ Aeromedical Center of Thessaloniki (AeMC), Kalamaria, Greece; ^4^ School of Computer Science, University of Lincoln, Lincoln, United Kingdom; ^5^ European Network of Living Labs, Brussels, Belgium; ^6^ Thirdage LLC., New York, NY, United States

**Keywords:** artificial gravity, cardiac output, deconditioning, graph theory, mean arterial pressure, short-arm human centrifuge, rehabilitation, exercise

## Abstract

**Introduction:** The idea behind the presentation of this case relates to utilizing space technology in earth applications with mutual benefit for both patients confined to bed and astronauts. Deconditioning and the progressiveness of skeletal muscle loss in the absence of adequate gravity stimulus have been of physiological concern. A robust countermeasure to muscle disuse is still a challenge for both immobilized patients and astronauts in long duration space missions. Researchers in the space medicine field concluded that artificial gravity (AG) produced by short-radius centrifugation on a passive movement therapy device, combined with exercise, has been a robust multi-system countermeasure as it re-introduces an acceleration field and gravity load.

**Methods:** A short-arm human centrifuge (SAHC) alone or combined with exercise was evaluated as a novel, artificial gravity device for an effective rehabilitation strategy in the case of a stroke patient with disability. The results reveal valuable information on an individualized rehabilitation strategy against physiological deconditioning. A 73-year-old woman was suddenly unable to speak, follow directions or move her left arm and leg. She could not walk, and self-care tasks required maximal assistance. Her condition was getting worse over the years, also she was receiving conventional rehabilitation treatment. Intermittent short-arm human centrifuge individualized protocols were applied for 5 months, three times a week, 60 treatments in total.

**Results:** It resulted in significant improvement in her gait, decreased atrophy with less spasticity on the left body side, and ability to walk at least 100 m with a cane. Balance and muscle strength were improved significantly. Cardiovascular parameters improved responding to adaptations to aerobic exercise. Electroencephalography (EEG) showed brain reorganization/plasticity evidenced through functional connectivity alterations and activation in the cortical regions, especially of the precentral and postcentral gyrus. Stroke immobility-related disability was also improved.

**Discussion:** These alterations were attributed to the short-arm human centrifuge intervention. This case study provides novel evidence supporting the use of the short-arm human centrifuge as a promising therapeutic strategy in patients with restricted mobility, with application to astronauts with long-term muscle disuse in space.

## 1 Introduction

### 1.1 The clinical problem

Stroke is the second most common cause of death worldwide and the most frequent cause of adult-onset and long-term acquired disability (Bonita et al., 2004; Lopez et al., 2006), leading to a harmful effect on the nervous, musculoskeletal, heart, lung, and gastrointestinal systems. Intracerebral hemorrhage (ICH) is a devastating form of stroke with a high mortality rate and poor prognosis (Mendelow et al., 2015). Nearly all the patients hospitalized with stroke suffer one or more medical or neurological adverse events (Langhorne et al., 2000; Ingeman et al., 2010). Almost 50% of deaths occurring in the first month after a stroke may be due to immobility-related adverse events (Lavados et al., 2007).

The use of gravity by means of long-term exercise combining aerobic and resistance training to enhance flexibility, balance, and coordination within daily activities for patients after a stroke has been recommended by the American Heart Association with specific guidelines published (Gordon et al., 2004). However, the restricted mobility of these patients and their inability to exercise properly lead to the need for alternative g-load rehabilitation techniques.

### 1.2 Strategies and evidence

Within 4 years of stroke onset, the resulting decreased physical activity (Gebruers et al., 2010) leads to fatigue within the first year (Duncan et al., 2012), and after 4 years of stroke onset, it leads to significant reductions with more than 30% evident in autonomy and in socializing participations (Gadidi et al., 2011). More than 75% of stroke patients receive rehabilitation therapy (Buntin et al., 2010), while many patients remain with residual functional disabilities, increasing the need for effective stroke care and the demand for individualized interventions (Mazrooyisebdani et al., 2018), targeted to improve stroke survivors’ lives by reducing disabilities and by regaining independence (Dobkin, 2005; Bindawas and Vennu, 2016).

Space researchers recognized early on that physical inactivity and gravitational demands on the body associated with bed rest, used to simulate the physiological conditions induced by the weightlessness of spaceflight, share the same mechanisms (Pavy-Le Traon et al., 2007). If gravity is not used due to inactivity or bed rest, it leads to negative consequences in all human physiological systems (Rittweger and Frings-Meuthen, 2013; McDonnell et al., 2019) since our health depends on gravity on Earth for optimal function (Vernikos, 1996; Vernikos et al., 1996; Vernikos, 1997; Vernikos, 2008; Vernikos, 2017).

The consequences of bed rest on human physiology led Space researchers to seek effective means of counteracting the detrimental effects of the lack of gravity in space. Several countermeasures have been tested (LeBlanc et al., 1988; Norsk, 2014). More recently, the intermittent administration of Gz combined with exercise using a short-arm human centrifuge (SAHC) was proposed as the most promising countermeasure for mitigating the physiological multisystem deconditioning in space or bed rest (Vernikos, 1997; Clement et al., 2015). This evidence-based medicine was introduced in stroke patients, since research in rehabilitation after stroke revealed that interventions with physical therapy and exercise provide additive value (Dobkin, 2005).

Stroke also affects bone mineral density (BMD) and lean tissue mass (Celik et al., 2008) in both legs, but the decline is more profound in the paretic lower limb (Beaupre and Lew, 2006). This, combined with balance deficits resulting from stroke, increases the risk of falls and fractures (Eng et al., 2008). Jorgensen and Jacobsen (2001) assessed 40 patients at 6 days, 7 months, and 1 year after stroke, and they reported that the changes in BMD after stroke are correlated with functional deficits in the paretic limb, strengthening the need for an effective individualized rehabilitation strategy. Additionally, brain injury resulting from unilateral stroke alters brain function and the complex balance within the cortical activity, affecting the functional network architecture of cortical areas in both hemispheres (Van Putten and Tavy, 2004; Grefkes et al., 2011; Grefkes and Fink, 2014).

Lipnicki et al. (2009) analyzed 16 articles on studies relating to the effects of bed rest on cognitive function and suggested that exercise may improve the molecular and cellular structure and function of the brain.

Stroke patients face difficulty in moving upright in the field of gravity and exercising properly, which led to the search for methods of passively receiving the gravity load, not unlike the need for astronauts to experience gravity in space. Space technology came to the rescue with a short-arm human centrifuge, where a gravity load imposed by centripetal acceleration may be used to protect effectively various physiological systems, including skeletal muscle and bone, the cardiovascular system, and the CNS (Baker, 2007). Exercise may have additional effects on brain cortical activity in a dose–response relationship between exercise mode and intensity, affecting regions according to task familiarization and adaptation (Brümmer et al., 2011).

In the stroke patient studied here, the effectiveness of AG was evaluated by means of multiple non-invasive physiological and EEG monitoring using a series of outcome measures of the musculoskeletal system (e.g., muscle strength, mobility, and balance test), providing an estimate of changes in muscle function and sensorimotor coordination (Van Putten and Tavy, 2004), on the cardiovascular system, such as SBP, DBP, MAP, cardiac output, stroke volume, and heart rate, and the neurological system, such as functional connectivity. We anticipated alterations in functional connectivity and cortical regions’ activations, especially in motor cortex regions and the regions affected by the stroke. Since physical training and aerobic exercise were associated with cortical reorganization within neuroplasticity-associated brain regions (Grefkes and Fink, 2011; Grefkes and Ward, 2014), we also anticipate enhancement of cortical excitability (Brümmer et al., 2011) due to the SAHC intervention. We would also focus on providing concrete evidence in support of “hypergravity therapy.” The latter may extend the applicability of the proposed training in aging diseases, such as bone atrophy (Morita et al., 2004) and ischemia (Sang et al., 2008).

## 2 Materials and methods

### 2.1 Patient report

This case study considered a 73-year-old right-handed female patient. Her weight was 72 Kg. Her height was 150 cm. She did not face any other comorbidities apart from high blood pressure, which was well controlled with medications. She experienced, 11 years ago, an intracerebral (intraparenchymal) hemorrhage which originated within the right thalamus and extended into the right lateral ventricle with cerebral edema and midline shift. The etiology of this hemorrhage was hypertension. At the timepoint of enrollment in the study, she was alert, attentive, and oriented with clear speech and fluent with repetition, comprehension, and naming. She did not experience dysphagia or dementia. The patient had a “scissors gait” and loss of normal arm swing on the left side with clenched fist and thumb in palm (Grade 4 modified Ashworth Scale) and atrophy of the left shoulder. Her left leg was in extension with plantar flexion of the foot and toes, weakness of distal muscles, and extensor hypertonia (Grade 4 modified Ashworth Scale). She also presented pyramidal signs on her left lower extremity with un-sustained clonus and absent plantar reflex. The patient was unable to walk 3 m even with a walking aid (cane). No botulinum toxin injections for spasticity were used, and only physiotherapy was administered. Pallesthesia (tuning fork 128 Hz) and exteroceptive sensation were decreased in the left extremities compared to the right. She was treated with human centrifugation (Kourtidou-Papadeli et al., 2021; Kourtidou-Papadeli et al., 2022) for five consecutive months, three times a week for half an hour, with an intermittent gravity treatment.

### 2.2 Ethics

The studies involving human participants were reviewed and approved by the Bioethics Committee of the School of Medicine of the Aristotle University of Thessaloniki (179/19.03.2020). The study protocol was registered on ClinicalTrials.gov (Identifier: NCT04369976). The patients/participants provided their written informed consent to participate in this study.

### 2.3 Experimental design

Intermittent centrifugation was selected for rehabilitation combined with exercise since it has greater beneficial effects on the musculoskeletal and the cardiovascular system (Iwase, 2005; Clément and Bukley, 2007; Edmonds, 2008; Paloski, 2014). Additionally, with the blood flow rushing towards the feet and contracting calf muscles to increase venous return, it prevents orthostatic intolerance (OI), thus decreasing the probability of pre-syncope during acceleration (Yang et al., 2007; Yang et al., 2010; Diaz Artiles et al., 2016). Among the symptoms of OI expected were nausea, sweating, confusion, dizziness, a narrowing of the visual field, an abrupt drop in MAP of > 20 mmHg, or a critical narrowing of the pulse pressure. Termination of centrifugation was determined when presyncope occurred either subjectively when the subject was feeling symptoms like nausea, sweating, gray out, omnidirectional vertigo, head vacuum feeling, dizziness, and sudden sensation of heat or objectively by the ClearSight non-invasive medical monitor when the following findings were detected: systolic blood pressure drop-off of 15 mmHg, decline of the heart rate of 15 beats/min suddenly, or the subject showing a sustainable high tachycardia. Additionally, the subject could press the panic button in case she wanted to stop.

The experimental design consisted of three phases.

Phase 1: Familiarization

Prior to her enrollment, the patient was submitted to a 10-min centrifugation trial at 1 g for familiarization. Since she tolerated the procedure without dizziness or nausea, she was enrolled in the study. She was advised to abstain from caffeine during training days, avoid alcohol and medications during the 12 h preceding the training session, avoid eating during the preceding 2 h, and avoid heavy exercise during the preceding 24 h. She was strapped to the centrifuge of a 2-m radius with safety harnesses, placing her head stable near the center of the centrifuge to avoid the Coriolis side effect due to centrifugation.

Phase 2: Initial evaluation training

The individualized protocols were evaluated during phase 2. During the initiation of this phase, we measured the cardiovascular parameters, specifically the cardiac output (CO) and mean arterial pressure (MAP), while the patient was in a standing position. Based on these values, we plotted dose–response curves that our team constructed in our previous paper (Kourtidou-Papadeli et al., 2021). Our aim was to get an estimation of the optimal g-load training for our patient. Then, the patient was submitted to the actual centrifugation with gradually increasing gravitational loads in order to verify the determination of the optimal gravity load for the 5-month treatment based on the cardiovascular parameters. The test started at .5 g, followed by .7, 1.0, 1.2, 1.5, 1.7, and 2 g, each for 5 min, with gradual acceleration and deceleration and with 6 min of pause between gravitational loads. We selected as the starting treatment intensity, the g-load with similar cardiovascular values to standing and close to the estimated value. This was the baseline treatment intensity for the patient. The onset acceleration was 0.2 g/s. The apex of the head was in touch with the rotation axis. We used this as the reference point to measure the distance to the feet and consequently the revolutions per minute needed to generate the appropriate g-load. There was a movable board adjusted to the soles of the feet to be in close contact with the board.

Phase 3: Rehabilitation treatment

The rehabilitation approach included two different protocols: A and B. Protocol A consisted of 1.7 g (6 min), 4 min break/1.7 g (6 min), 4 min break/1.7 g (6 min), 4 min break/1.7 g (6 min), 4 min break/2 g (6 min), and protocol B consisted of 2 g (10 min), 4 min break, 2.2 g (10 min), 4 min break, 2.5 g (10 min) with SAHC alone and the same protocols combined with exercise, resulting in five conditions as pre-SAHC, protocol A, protocol A + exercise, protocol B, and protocol B + exercise of 5 weeks. Each condition included 15 sessions of centrifugation, with measurements of the strength and balance taken after 5 min standing for “pre” and 5 min after the end of centrifugation for “post.” The EEG data were taken before entering the rehabilitation with SAHC and after each protocol. The total rehabilitation treatment was 5 months. Each measurement of grip strength, posturography, and balance was performed three times, and the best effort was accepted. The protocol’ description is presented in Table 1.

**TABLE 1:** Different protocols applied on SAHC.

Protocols SAHC	Centrifugation loads and exercise
Protocol A	1.7 g (6 min), 4 min break/1.7 g (6 min), 4 min break/1.7 g (6 min), 4 min break/1.7g (6 min), 4 min break/2 g (6 min)
Protocol A + exercise	1.7 g (6 min), 4 min break/1.7 g (6 min), 4 min break/1.7 g (6 min), 4 min break/1.7 g (6 min), 4 min break/2 g (6 min) + bicycle + arm/upper limbs resistance
Protocol B	2 g (10 min), 4 min break, 2.2 g (10 min), 4 min break, 2.5 g (10 min)
Protocol B + exercise	2 g (10 min), 4 min break, 2.2 g (10 min), 4 min break, 2.5 g (10 min) + bicycle + arm/upper limbs resistance

Each training session consisted of a total of 30 min intermittent centrifugation with 4 min pause in between. The total time of the session (including preparatory and recovery steps) was approximately 1 h and a half, and the training frequency was three times per week.

Each protocol lasted 5 weeks with a total rehabilitation time of 5 months. During centrifugations, cardiovascular and electroencephalographic (EEG) responses were continuously monitored. Before and after the 5-month rehabilitation program, neurological and physical performance tests were also carried out. Additionally, before each centrifugation session, balance and muscle strength were assessed.

During rotation, the participant’s eyes were covered with an eye cover, and lights were off to remove visual cues. Then, she was instructed to avoid moving her head to avoid the Coriolis effect.

In order to safely use the centrifuge, the participant was required to fulfill some requirements. She was healthy, with a height not exceeding 2 m, and with impaired mobility from a stroke. She did not have any neurological or psychiatric disorder, vertigo, nausea or chronic pain, chronic use of substances or alcoholism, recent (within 6 months) surgery, current arrhythmia, severe migraines, pregnancy, epilepsy, chololithiasis or kidney stones, dehydration, recent wounds from surgery, recent fractures (unless recommended by a doctor), acute inflammation or pain, and newly inserted metal pins or plates or newly implanted stents.

### 2.4 The human centrifuge

#### 2.4.1 Theoretical concept

SAHC is an integrated multi-system countermeasure in order to provide artificial gravity (AG) loads for rehabilitation purposes in case of physiological deconditioning due to inactivity or lack of gravitational load in the Gz direction. The AG functions by exerting a centrifugal force on a body, accelerated centripetally in a rotating device (Vernikos, 1996; Yang et al., 2010). The participant lies in a supine and horizontal position on the rotation bed, with the head towards the center. The patient is forced away from the axis of rotation with a force that is the product of body mass, distance from the axis of rotation, and angular velocity squared. This force can be calculated from the equation F = mrw^2^, where m is the mass of the subject, r is the distance from the center of the centrifuge to the center of mass of the subject, and w is the velocity at which the bed rotates. Thus, the force exerted at the feet increases with the velocity of rotation and with the body’s distance from the axis of rotation. The reference point for the g-level applied is the feet of the subject. The head of the participant is aligned 10 cm close to the center of centrifuge rotation. So, when regarding a subject of 1.70 m height (+10 cm of the distance to the top of the head = 1.80 m) with a gravity gradient of 1.0 g (23 rotations/minute) at the feet, the g-load on top of the head is .06. This design greatly minimizes a Coriolis side effect, while the g-load on the feet may reach 3.5 *g* at the maximal velocity of the device. Moreover, the AG level plays a role in the magnitude of the Coriolis effect, as well as the patient’s head movement, since Coriolis force also derives from the mismatch of vestibular information and the effect of Earth’s gravity.

#### 2.4.2 The short-arm human centrifuge architecture

The short-arm human centrifuge (SAHC) was developed by our research group (patent #1009812/13/09/2019) and is located at the “Joan Vernikos” Laboratory of Aerospace and Rehabilitation Applications. The diameter is 4 m, with the possibility of reaching a gravity load at the feet of +3.5 Gz. It consists of a support base: a central axis with two beds connected and rotating smoothly on wheels. Our previous experiments validated the mechanical aspects of the centrifuge and the successful attainment of physiological data during centrifugation and exercise. Subjects with neurodegenerative diseases well tolerated the centrifugation and successfully completed the exercise protocol.

#### 2.4.3 Aerobic exercise intervention

The centrifugation on the SAHC was combined with mild-intensity exercise based on both percentage of predicted maximal heart rate (%PMHR) and the Karvonen formula (Karvonen et al., 1957). Mild-intensity exercise includes 40%–59% maximal heart rate (MHR) and 30%–49% Karvonen, corresponding to 5–8 metabolic equivalents (METS) (Powers and Howley, 1997, p.292; Wilmore and Costill, 1994, p.524, deVries and Housh, 1994, p.297). Karvonen formula was a reasonably accurate method for estimating exercise intensity, as demonstrated by Davis and Convertino (1975). Both methods seemed to be similar. The subjects were monitored during centrifugation using a Bluetooth Polar H10 device with an accurate heart rate sensor, and the intensity of the exercise was kept at “light intensity”. The subject was continuously monitored and advised accordingly to ensure the exercise pace remained within the predetermined limits.

#### 2.4.4 Aerobic exercise infrastructure

The artificial gravity training is further enhanced through a state-of-the-art aerobic exercise infrastructure. It consists of a bicycle ergometer, feet resistance, and arm/upper limb elastic band resistance.

Removable exercise equipment was attached to the bed extremities with the possibility of aerobic training through a bicycle ergometer adjusted at the feet according to the patient’s height and resistance training through a horizontal rowing device for the lower limbs with elastic bands attached to the axis of the device for the arm/upper limbs. It has been constructed and evaluated in healthy individuals according to both national and international safety regulations, while also being applicable to patients with mobility disabilities.

### 2.5 Neurological/medical description

The Berg Balance Scale (BBS) was used for estimating the fall risk of the patient (Blum and Korner-Bitensky, 2008). This balance score is associated with quality of life (Schmid et al., 2013) and is a sensitive (80%) and specific (78%) tool for identifying individuals at risk of falling following a stroke (Maeda et al., 2009).

### 2.6 Electroencephalographic analysis

#### 2.6.1 Data acquisition

The data acquisition was performed in three experimental time instances: before SAHC, SAHC alone in both protocols, and SAHC combined with exercise on the resting state (eyes closed) condition. The patient was instructed to close her eyes and remain calm for approximately 5 min. In case there were artifacts due to body movements, opening of eyes, or a muscle/body movement, the recording session was extended proportionally. The room temperature was kept at a relatively stable condition (22°C) through artificial cooling, and the room lights were turned off. The operators also kept auditory noise minimal.

The EEG device was the 32-channel Neurofax EEG-1200 connected to a laptop (Nihon Kohden, Tokyo, Japan). Among those electrodes, there were 1) 19 EEG electrodes placed according to the 10–20 International System, 2) two electromyographic/EMG electrodes for recording chin movements, 3) two electrodes for electroculogrammic/EOG (both horizontal and vertical) recordings, and finally, 4) two electrocardiographic/ECG electrodes. Apart from the electrodes used for EEG data, all the others (ECG, EMG, and EOG) were bipolar. The ground electrode was placed on the prefrontal midline (Fpz) position. There were also two reference electrodes placed on the left and right mastoid muscles. The sampling frequency was kept at 500 Hz for all the signals.

#### 2.6.2 EEG Preprocessing

This step was performed through custom scripts on Python 3.6. First, we performed a common average re-referencing. Then, we applied several Butterworth filters of second order with the following order:

• High-pass filter with the cut-off frequency at .5 Hz
• Low-pass filter with the cut-off frequency at 50 Hz
• Three band-stop (notch) filters centered at 50, 100, and 150 Hz, respectively

The high-pass filter was used to remove DC shifts (linear trends). The low-pass filter was used for removing high-frequency noise. The first band-stop filter was used for removing the industrial noise induced by the power supply, and the other ones were for removing the industrial noise harmonics. Then, the filtered data were subjected to independent component analysis as implemented through the EEGLAB graphical user interface under the MATLAB environment. Two experienced neuroscientists inspected the data in order to recognize and remove artifactual sources. Then, visual inspection was also performed on the electrodes’ time series to remove time segments that were still heavily contaminated with noise. The remaining data were divided into non-overlapping epochs. The epoch duration was 16 s.

#### 2.6.3 Cortical functional connectivity analysis

The analysis was performed through the Brainstorm graphical user interface and custom scripts in MATLAB. The Brainstorm was used for modeling the generic head anatomy by employing the M/EEG boundary element method. We employed the default Brainstorm settings for modeling the cortex by means of 15,000 dipoles of fixed orientation. The estimation of the cortical activity was performed by solving the inverse problem through the sLORETA methodology as implemented within the Brainstorm software. Then, we grouped the 15,000 dipoles into 148 cortical regions according to the Destrieux atlas (Destrieux et al., 2010). This is a probabilistic sulco–gyral atlas of the human cerebral cortex. The cortical activity of each region was estimated as the mean value of all the dipoles consisting of that region. Then, we performed functional connectivity analysis through the synchronization likelihood/SL method (Stam and Van Dijk, 2002). This method was selected since it is superior to linear and symmetric (coherency) metrics by avoiding the bias of the freedom degrees and its robustness when dealing with non-stationary dynamics (Montez et al., 2006). Its output is a 148 × 148 connectivity matrix. Each cell of this matrix denotes the co-operative degree among the given pair of cortical regions. The values range from 0…1. Lower values denote minimal connectivity among the specific pair, whereas larger values denote stronger co-operative activity. The main diagonal line is equal to 1, since each region is compared with itself.

#### 2.6.4 Cortical network analysis

The analysis was performed through custom MATLAB scripts, which employed functions derived from the Brain Connectivity Toolbox (BCT). We used binary, non-directed graphs by detecting the 20% largest pairs of connectivity values. This was achieved by applying an adaptive threshold for each epoch to ensure that all the graphs were of the same density. The selected edges were binarized to “1”, while all the other pairs of the connectivity matrix were set as “0”. Then, we estimated global network metrics such as the mean cluster coefficient, the characteristic path length, the global efficiency score, and the modularity score. Since we were interested in the identification of the most prominent hubs, we also estimated the normalized betweenness centrality metric.

Then, we computed the following network metrics:

• Node degree: It is the number of edges that each node contains.
• Distance matrix: It is computed as the shortest path among two nodes. Therefore, it is the minimum number of edges needed to reach from one node to any other.
• Characteristic path length: It is the mean value of the shortest paths among all node pairs.

### 2.7 Cardiovascular analysis

#### 2.7.1 Cardiovascular measures

**FIGURE 1:**
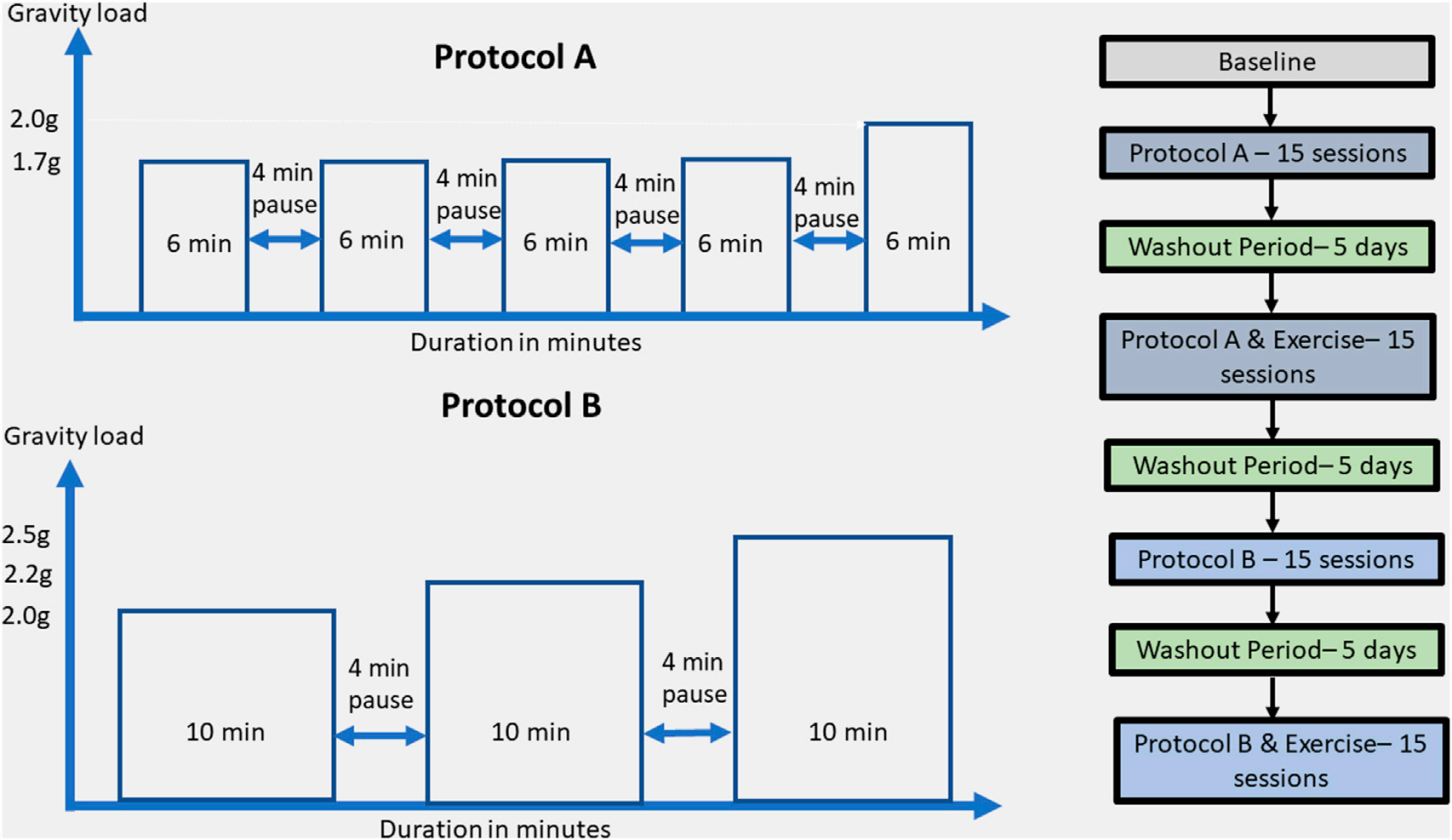
Detailed description of the protocol A and B training components on the upper left and right side, respectively. The entire rehabilitation approach in terms of protocols, exercise presence or not, wash-out periods, and number of sessions is described in the right part of the figure.

Systolic blood pressure (SBP) mmHg, diastolic blood pressure (DBP) mmHg, mean arterial pressure (MAP) mmHg, cardiac output (CO) L/min, stroke volume (SV) mmHg, and heart rate (HR) were monitored continuously and non-invasively with finger plethysmography with the ClearSight device (Edwards Life Sciences) (Figure 1).

### 2.8 Muscle analysis

#### 2.8.1 Grip strength

**FIGURE 2:**
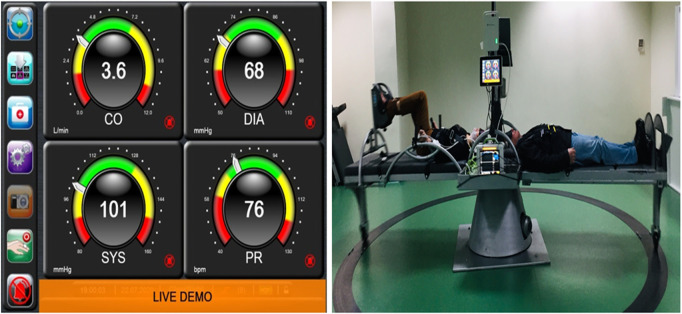
Finger plethysmography (left side) instant values of cardiovascular parameters monitored by the ClearSight device connected to the patient during centrifugation and recording data every 30 s. SAHC with the patients on the beds, one at the left bed with bicycle ergometer and the other with SAHC only and simultaneous recordings is visualized on the right side of the screen.

The outcome measures derived from this device are max. strength % (MS%), max. strength in kilograms (MSKg), and max. strength deficit % (MSD%). Grip strength has been found to be a useful objective measure of motor impairment, particularly the rate of increase in grip forces (Renner and Bungert-Kahl, 2009). To evaluate, reassess, and monitor grip strength, the K-FORCE grip dynamometer developed by KINVENT company was used, which works *via* Bluetooth Android application (Figure 2).

#### 2.8.2 Posturography and balance

Static and dynamic posturography, weight distribution difference (WDD), average position of the center of pressure (APCOP) in the medio-lateral and antero-posterior planes, and average velocity of displacement (AV) were evaluated with a frequency of up to 75 Hz. These data were processed with KINVENT’s Balance Clinic software (Figure 2).

### 2.9 Statistical analysis

For Statistical analysis, Python 3.8.5 and R Studio 1.3.1093 were used on a Windows PC. The level of statistical significance was set to *p* < .05. For continuous variables, we calculated descriptive statistics (through tables and boxplots), means, and standard deviations, and for categorical variables, absolute and relative frequencies were calculated. One-way, repeated measures analyses of variance (ANOVAs) were conducted for each independent categorical variable. When sphericity was not assumed, Greenhouse–Geisser corrections were applied.

## 3 Results

### 3.1 Cortical network characteristics

The measurements were performed in three-time experimental instances: before SAHC (Pre), SAHC alone in both protocols (mid), and SAHC combined with exercise (post). The analysis was focused on the right side of the brain, which affected the left side of the patient.

#### 3.1.1 Right postcentral gyrus

The descriptive statistics of the network characteristics for the right postcentral gyrus in the various time instances are reported in Table 2:

**TABLE 2:** Descriptive statistics for the right postcentral gyrus, where “pre” stands for before SAHC, “mid” stands for SAHC alone in both protocols, and “post” stands for SAHC combined with exercise.

Network metric	Mean value ± standard deviation			Minimum value			Maximum value		
	Training phase			Training phase			Training phase		
	Pre	Mid	Post	Pre	Mid	Post	Pre	Mid	Post
Cluster	.636 ± .063	.438 ± .074	.478 ± .074	.504	.318	.364	.775	.632	.683
Betweenness centrality	.420 ± .237	1.265 ± .891	1.880 ± .956	.085	.399	.615	.983	3.105	3.644
Node degree	70.889 ± 13.164	65.333 ± 18.111	90.111 ± 17.129	50	38	68	98	104	118

There was a statistically significant main effect of the intervention on the 1) cluster coefficient F (1,34) = 64.953, *p* < .0001, 2) betweenness centrality metric F (1,34) = 17.975, *p* < .0001, and 3) node degree F (1,34) = 11.391, *p* = .0002.

*Post hoc* comparisons performed regarding the Cluster coefficient identified a statistically significant difference between pre and mid values (t = 10.708, *p* < .001) and pre and post values (t = 13.360, *p* < .001) and a marginal significance between mid and post values (t = -1.746, *p* = .099).

*Post hoc* comparisons performed regarding the betweenness centrality metric identified a statistically significant difference between pre and mid values (t = −4.071, *p* = .001) and pre and post values (t = −7.320, *p* < .001) and a marginal significance between mid and post values (t = −1.98, *p* = .064).

*Post hoc* comparisons performed regarding the node degree did not identify a statistically significant difference between pre and mid values (t = .964, *p* = .349). However, there was a statistically significant difference between pre and post values (t = −5.149, *p* < .001) and between mid and post values (t = −3.830, *p* = .001).

#### 3.1.2 Right precentral gyrus

The descriptive statistics of the network characteristics for the right precentral gyrus in the various training phases are reported in Table 3:

**TABLE 3:** Descriptive statistics for the right precentral gyrus, where “pre” stands for before SAHC, “mid” stands for SAHC alone in both protocols, and “post” stands for SAHC combined with exercise.

Network metric	Mean value ± standard deviation			Minimum value			Maximum value		
	Training phase			Training phase			Training phase		
	Pre	Mid	Post	Pre	Mid	Post	Pre	Mid	Post
Cluster	.596 ± .077	.503 ± .082	.559 ± .079	.439	.385	.392	.772	.693	.708
Betweenness centrality	.633 ± .436	.870 ± .499	1.344 ± .724	.100	.218	.488	1.803	1.988	3.002
Node degree	72.222 ± 14.996	67.667 ± 18.436	96 ± 17.026	46	38	64	100	102	138

There was a statistically significant main effect of the intervention on the 1) cluster coefficient F (1,34) = 6.976, *p* = .0029, 2) betweenness centrality metric F (1,34) = 6.88, *p* = .003, and 3) node degree F (1,34) = 13.529, *p* < .0001.

*Post hoc* comparisons performed regarding the cluster coefficient identified a statistically significant difference between pre and mid values (t = 4.085, *p* = .001), no statistical significance between pre and post values (t = 1.460, *p* = .163), and a marginal significance between mid and post values (t = −2.081, *p* = .053).

*Post hoc* comparisons performed regarding the betweenness centrality metric did not show a statistically significant difference between pre and mid values (t = −1.640, *p* = .119). There were statistically significant differences between pre and post values (t = −3.167, *p* = .006) and between mid and post values (t = −2.286, *p* = .035).

*Post hoc* comparisons performed regarding the node degree did not show a statistically significant difference between pre and mid values (t = .778, *p* = .447). There were statistically significant differences between pre and post values (t = −4.471, *p* < .001) and between mid and post values (t = −4.477, *p* < .001).

**FIGURE 3:**
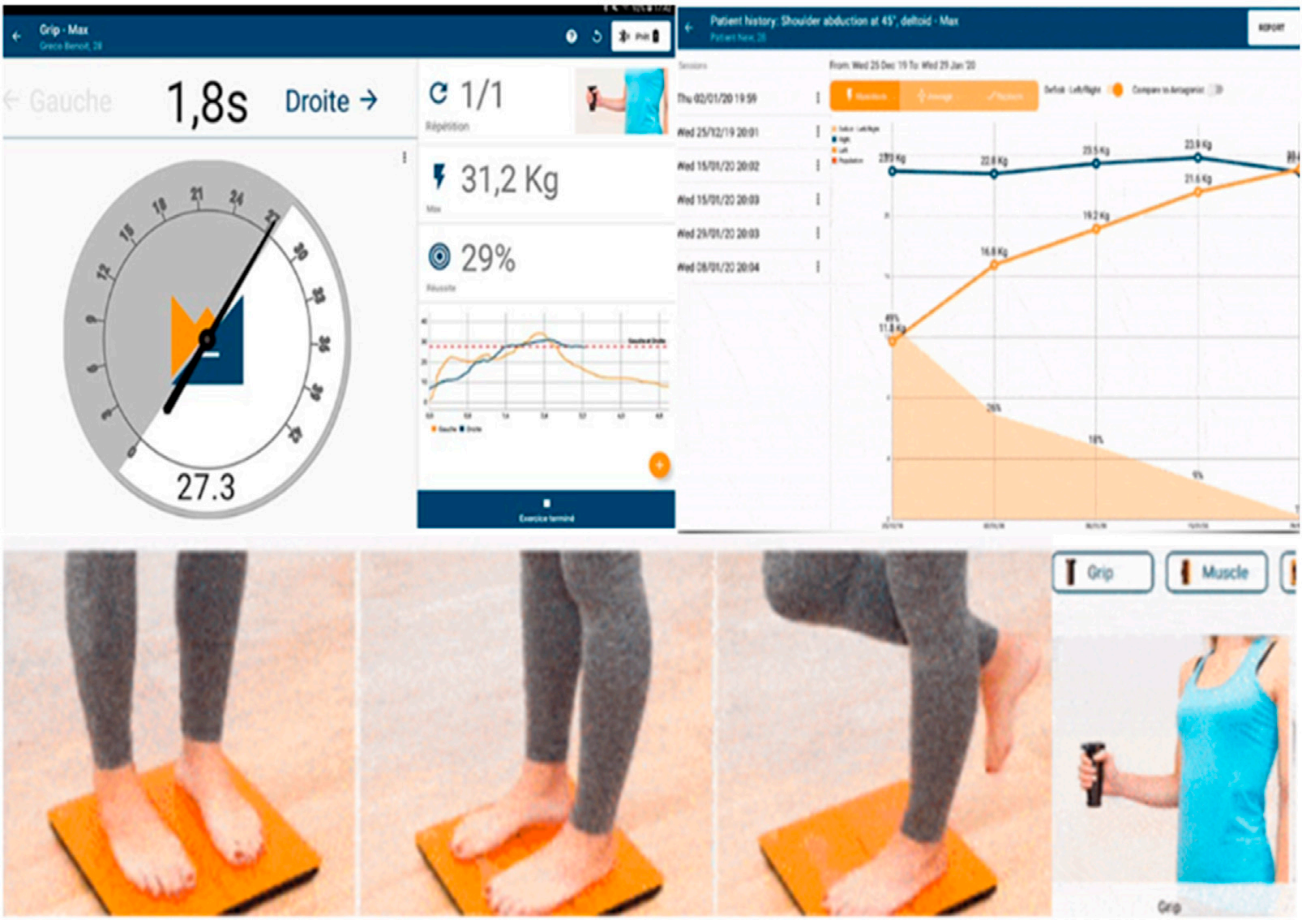
Max. grip strength dynamometer assessment (patient at the right lower corner) with the results at the left upper corner and the improvement of the studying period. Posturography K-FORCE plates, as seen at the left lower corner, for posture and stance evaluation, center of pressure, and weight distribution measurement.

The differences for both cortical regions are visualized in Figure 3.

### 3.2 Muscle analysis results

Table 4 visualizes the statistically significant main effects for the various independent variables regarding the muscle analysis. The muscles that are involved are the grip muscles, and among them are the flexor digitorum profundus, flexor digitorum superficialis, flexor digiti minimi brevis, flexor pollicis longus, extensor digitorum, lumbricals, interossei, and adductor pollicis. The measurements performed were the 1) maximum strength percentage, 2) maximum strength, 3) weight distribution percentage, 4) total range, 5) maximum strength deficit (Percentage), 6) total CoP displacement, and 7) average velocity. The maximum strength percentage, maximum strength percentage, and weight distribution percentage were analyzed on the left and the right body sides, and the total range was analyzed on lateral and longitudinal positions, while the remaining ones (maximum strength deficit, total CoP displacement, average velocity) were analyzed. Then, we performed Tukey *post hoc* comparisons for the independent variables that induced either a main effect or an interaction effect.

**TABLE 4:** Visualization of the main effects of protocol, exercise, and their interaction on the various muscle variables.

Independent variable	Protocol		Exercise		Protocol × exercise	
	Left	Right	Left	Right	Left	Right
Maximum strength percentage	F (1,56) = 13.795, *p* = .0005	F (1,56) = 17.245, *p* = .0001	F (1,56) = 25.124, *p* < .0001	F (1,56) = 29.176, *p* < .0001		
Maximum strength		F (1,56) = 25.138, *p* = <.0001	F (1,56) = 23.267, *p* = <.0001			F (1,56) = 11.800, *p* = <.0011
Weight distribution percentage			F (1,56) = 4.66, *p* = <.0352			
	Lateral	Longitudinal	Lateral	Longitudinal	Lateral	Longitudinal
Total range				F (1,56) = 8.156, *p* = <.006		
Maximum strength deficit (percentage)	F (1,56) = 14.120, *p* = <.0004		F (1,56) = 24.562, *p* = <.0001			
Total CoP displacement						
Average velocity			F (1,56) = 6.192, *p* = <.0158			

**FIGURE 4:**
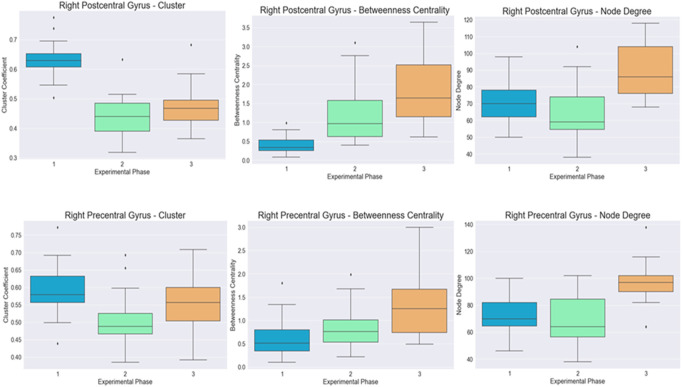
Visualization of the distribution of the cluster coefficient, betweenness centrality, and node degree for the right postcentral and right precentral gyrus across the three-time instances during the experimental phase, where “1” stands for before SAHC, “2” stands for SAHC alone in both protocols, and “3” stands for SAHC combined with exercise. For clarification, both AG alone or if combined with exercise is considered treatment. So, the only “pre” period is before centrifugation, and every protocol after that stands for “post.”

**FIGURE 5:**
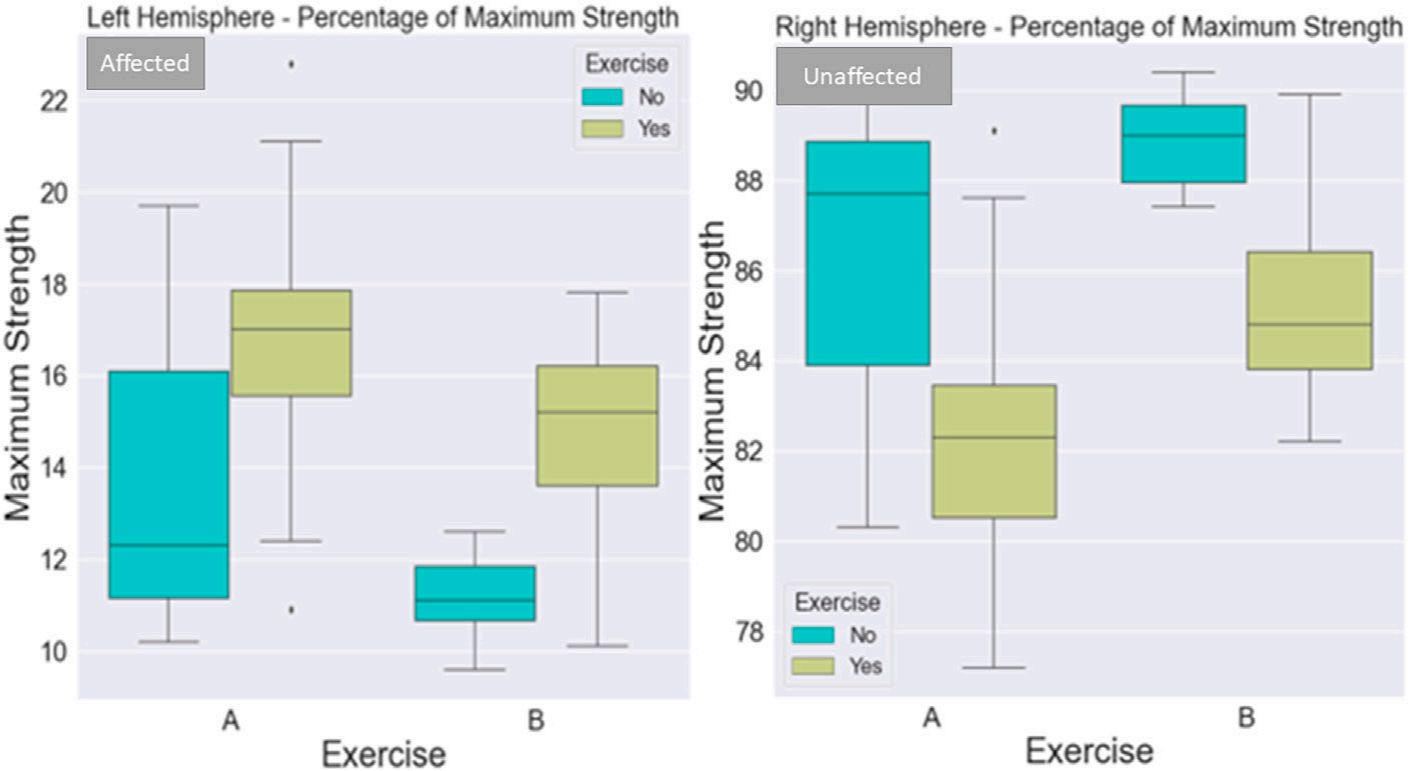
Visualization of maximum strength percentage for the left and right sides of the body. The maximum strength of the affected side (left part of the image) improved with exercise in both protocols. The maximum strength of the non-affected side (right part of the image) improved with both protocols when not combined with exercise. The exercise added to the centrifugation was very light to make a difference for the unaffected side.

Tukey *post hoc* comparisons for the independent variables induced either a main effect or an interaction effect. The results are reported in the following sub-sections and visualized in Figures 4, 5.

#### 3.2.1 Maximum strength percentage

When considering the left side of the body, there was a statistically significant difference between 1) A and B protocols when there is no exercise (*p* = .036) (Figure 4), 2) regarding the A protocol between no exercise and exercise existence (*p* = .0069) (Figure 4), 3) protocol A with exercise vs. protocol B with no exercise (*p* < .0001) (Figure 4), 4) protocol B with and without exercise (*p* = .003) (Figure 4), and 5) marginally statistically significant differences regarding protocol A and protocol B with exercise (*p* = .08).

When considering the right side of the body, there was a statistically significant difference between 1) A and B protocols when there is no exercise (*p* = .047) (Figure 4), 2) regarding the A protocol between no exercise and exercise existence (*p* = .0008) (Figure 4), 3) protocol A with exercise vs. protocol B with no exercise (*p* < .0001) (Figure 4), 4) protocol B with and without exercise (*p* = .004), and 5) protocol A and protocol B with exercise (*p* = .012).

#### 3.2.2 Maximum strength

When considering the left side of the body, a statistically significant difference regarding 1) the A protocol between no exercise and exercise existence (*p* = .0003) (Figure 5), 2) protocol A with exercise vs. protocol B with no exercise (*p* = .0002) (Figure 5), and 3) marginal statistically significant difference regarding the protocol B with and without exercise (*p* = .082) (Figure 5).

When considering the right side of the body, there was a statistically significant difference between 1) A and B protocols when there is no exercise (*p* < .0001) (Figure 5), 2) marginal statistically significant difference regarding the A protocol between no exercise and exercise existence (*p* = .077), 3) protocol B with exercise vs. protocol A with no exercise (*p* = .004) (Figure 5), 4) protocol A with exercise vs. protocol B with no exercise (*p* = .005) (Figure 5), and 5) marginal statistically significant difference regarding the protocol B with and without exercise (*p* = .09).

#### 3.2.3 Weight distribution percentage

When considering the left side of the body, there was a marginally statistically significant difference regarding protocol A, with versus without exercise (*p* = .086). Since there were no statistically significant differences in the right side of the body, no *post hoc* comparisons were performed.

#### 3.2.4 Total range

Since there were no statistically significant differences regarding the lateral parameter, no *post hoc* comparisons were performed.

When considering the longitudinal parameter, there was a statistically significant difference regarding protocol A, with versus without exercise (*p* = .040), and protocol A with versus protocol B without exercise (*p* = .024).

#### 3.2.5 Maximum strength deficit (percentage)

There was a marginal statistically significant difference between protocols A and B without exercise (*p* = .055). There was a statistically significant difference regarding protocol A with versus without exercise (*p* = .004). There was a statistically significant difference between protocol A with exercise versus protocol B without exercise (*p* < .001). There was a statistically significant difference regarding protocol Β with versus without exercise (*p* = .006). There was a marginal statistically significant difference between protocols A and B with exercise (*p* = .043).

#### 3.2.6 Total CoP displacement

Since there were no statistically significant differences for this feature, no *post hoc* comparisons performed.

#### 3.2.7 Average velocity

There was a statistically significant difference regarding protocol Β with versus without exercise (*p* = .049).

### 3.3 Cardiovascular analysis results

**FIGURE 6:**
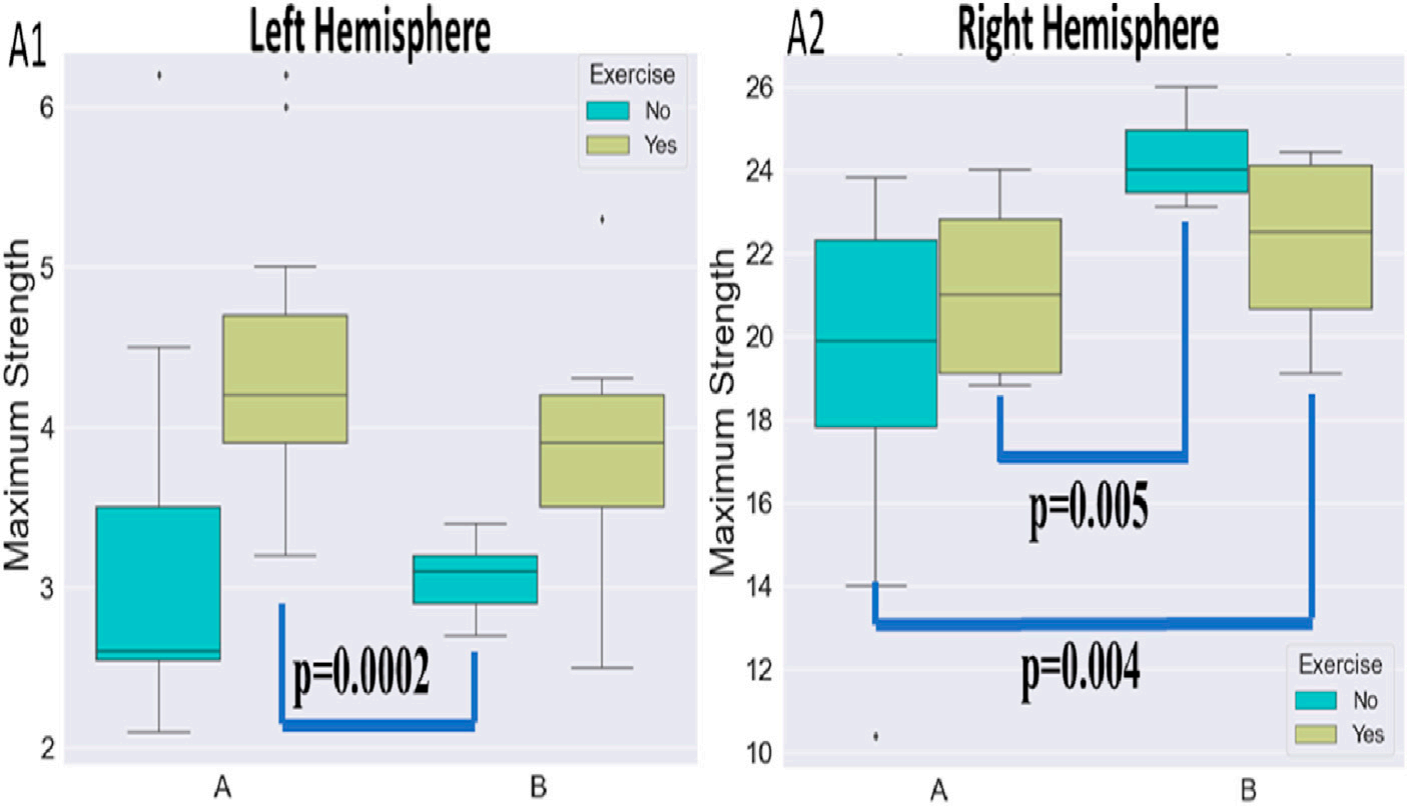
Visualization of maximum strength alterations for the left **(A1)** and right **(A2)** hemisphere and the various protocols.

Since our analysis aims to investigate the impact of two independent variables (both protocol and exercise type) on several cardiac markers, we wished to detect either a significant main effect of each variable or an interactive one. Therefore, we chose to visualize the distribution of the cardiac markers (A: cardiac output, B: cardiac index, C; stroke volume, D: pulse rate, E: mean arterial pressure, and F: systolic pressure) during the various exercise conditions through two different graphs (Figure 6).

**FIGURE 7:**
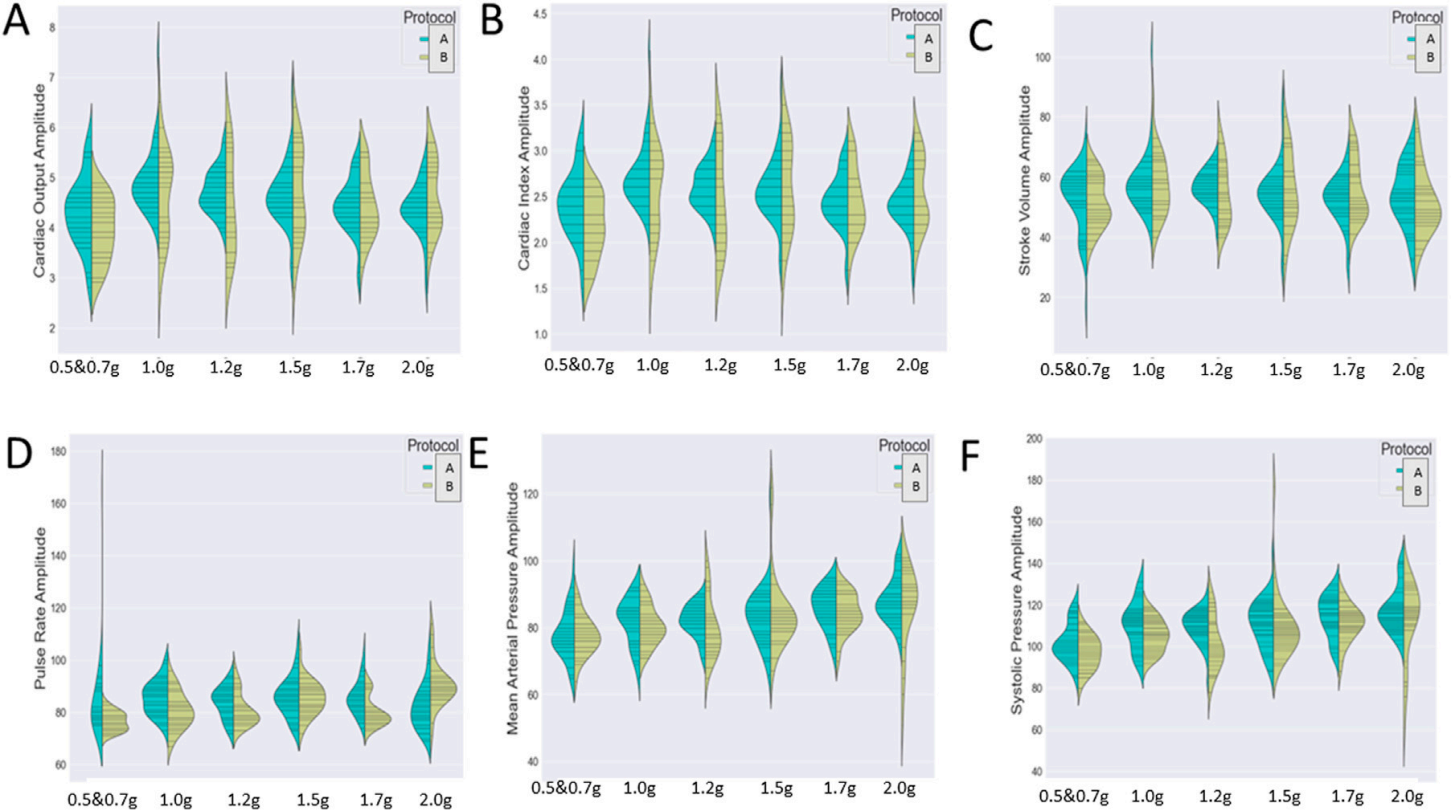
Visualization of six cardiac markers [**(A)** cardiac output, **(B)** cardiac index, **(C)** stroke volume, **(D)** pulse rate, **(E)** mean arterial pressure, and **(F)** systolic pressure] comparing the effect of protocols A (1) and B (2) either combined with exercise or not. The number of measurements for SAHC alone (both protocols) was 30 measurements (three measurements per week), and that for combined with exercise (both protocols) was also 30 measurements. For the grip strength and balance, the measurements were taken upon arrival after 5 min standing for the “pre” values and 5 min after the end of centrifugation for the “post” values. The EEG data were taken before entering the rehabilitation with SAHC and after each protocol. The figure visualizes the distribution of each marker’s instance for AG training protocol A alone and combined with exercise (green color) and protocol B alone and combined with exercise (yellow color). Elongated violin plot edges represent the existence of outliners, while their width is proportional to the existence of the instances.

**FIGURE 8:**
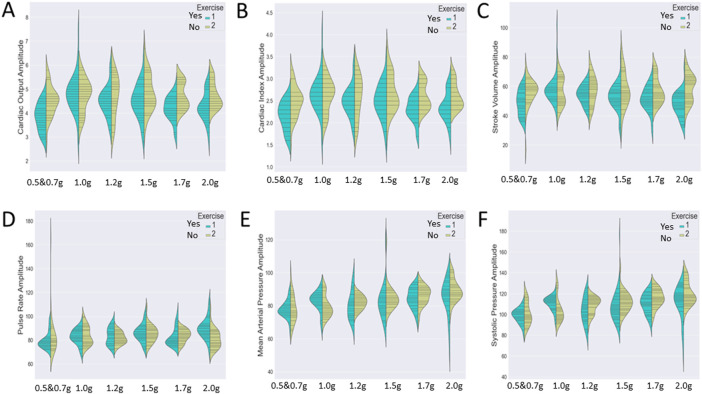
Visualization of six cardiac markers [**(A)** cardiac output, **(B)** cardiac index, **(C)** stroke volume, **(D)** pulse rate, **(E)** mean arterial pressure, and **(F)** systolic pressure] comparing the effect of exercise, independently of the protocol. The number of measurements for SAHC alone (both protocols) was 30 measurements (three measurements per week), and that for combined with exercise (both protocols) was also 30 measurements. For the grip strength and balance, the measurements were taken upon arrival after 5 min standing for the “pre” values and 5 min after the end of centrifugation for the “post” values. The figure visualizes the distribution of each marker’s instance for AG training protocols A and B together with no exercise (1) (green color) and protocols A and B together with exercise (2) (yellow color) through ergometer and resistive exercise of arm/upper limb. Elongated violin plot edges represent the existence of outliners, while their width is proportional to the existence of the instances.

Figure 7 represents the variation of the cardiac markers according to the different protocols (1: protocol A, 2: protocol B), while Figure 8 represents the impact of exercise (1: no exercise; 2: exercise existence). So, Figure 7 merges the exercise type and compares protocol A with or without aerobic exercise versus protocol B with or without exercise. Both Figures 7, 8 contain six violin plots, each one corresponding to the six SAHC g-loads (1: lying, 2: 1.5 g, 3: 1.7 g, 4: 1.7 g, 5: 1.7 g, and 6: 1.7 g) which are part of the proposed intervention procedure. On the other hand, we compare both protocols without exercise versus both protocols with exercise in Figure 8. Although boxplots are much more frequent in biomedical research studies, we selected violin plots since they are much more informative. Box plots contain the summary statistics, whereas violin plots visualize the entire data distribution. So, it is of particular importance to understand the peak distribution and the existence of potential outliers.

There was a statistically significant *protocol × exercise × condition* triple interaction for *pulse rate*: [F (1,388) = 4.055, *p* = .04473].

There was a statistically significant *protocol × condition* double interaction for 1) *cardiac output*: [F (1,388) = 8.827, *p* = .0032], 2) *cardiac index*: [F (1,388) = 10.294, *p* = .00145], and 3) *pulse rate*: [F (1,388) = 24.699, *p* < .0001].

There was a statistically significant *exercise × condition* double interaction for 1) *stroke volume*: [F (1,388) = 5.055, *p* = .0251], 2) *stroke volume index* [F (1,388) = 5.277, *p* = .0221], 3) *pulse rate* [F (1,388) = 8.947, *p* < .00296], 4) *systolic pressure*: [F (1,388) = 8.518, *p* = .004], and 5) *mean arterial pressure*: [F (1,388) = 4.430, *p* = .0359].

There was a statistically significant *protocol × exercise* double interaction for 1) *systolic pressure:* [F (1,388) = 4.971, *p* = .02635] and 2) *mean arterial pressure* [F (1,388) = 4.685, *p* = .0310].

There was a statistically significant main effect of exercise for 1) *cardiac output*: [F (1,388) = 24.803, *p* < .0001], 2) *cardiac index*: [F (1,388) = 22.830, *p* < .0001], 3) *stroke volume*: [F (1,388) = 27.460, *p* < .0001], 4) *stroke volume index*: [F (1,388) = 27.825, *p* < .0001], and 5) *systolic pressure*: [F (1,388) = 5.732, *p* = .0171].

There was a statistically significant main effect of protocol for 1) *pulse rate*: [F (1,388) = 7.286, *p* = .00725] and 2) *systolic pressure* [F (1,388) = 17.728, *p* < .0001].

There was a statistically significant main effect of condition for 1) *pulse rate*: [F (1,388) = 9.629, *p* = .00206], 2) *systolic pressure*: [F (1,388) = 71.306, *p* < .0001], 3) *diastolic pressure*: [F (1,388) = 29.845, *p* < .0001], and 4) *mean arterial pressure*: [F (1,388) = 58.771, *p* < .0001].

### 3.4 Neurological evaluation results

The Berg Balance Scale (BBS) score of the patient, when enrolled in the study, was = 6, which is interpreted as almost 100% fall risk and the patient is either already wheelchair-bound or may be soon. The improvement of the BBS after the treatment was 26, while the patient now may require assistance in performing certain activities of daily living, such as walking.

Somatosensory impairments: the patient presented somatosensory impairments of both left upper and lower limbs, with no sensory reaction before rehabilitation and started feeling the paretic side in the first month of rehabilitation; she could feel and move the upper and lower limb in the second month, could stand with no cane in the third month, and could walk several steps without a cane at the end of the treatment period of 5 months. After centrifugation, the gait was much better with less spasticity (no scissors gait) on the left body side. There was still no arm swing, and the spasticity of the left fist was better with no thumb in palm. There was a decrease in left shoulder atrophy. Pyramidal signs of the left lower extremity remained (unsustained clonus and absent plantar reflex). The patient, after a 5-month treatment period, was able to walk 100 m with a cane.

## 4 Discussion

The main finding of this study was the effectiveness of the SAHC alone or combined with aerobic and resistive exercise on all cardiovascular parameters, on strength and functional activities of both paretic and normal upper limb, as well as posturography and balance, leading to a significant decrease in disability. When the centrifuge was acting alone, then the protocol played a significant role. When protocols were combined with exercise, both were effective. The EEG graph theory analysis revealed neuroplasticity with significant improvement after treatment in the areas of the right precentral gyrus and right postcentral gyrus areas.

Hemiplegia and, consequently, the deterioration of motor skills play a major role in the reduction of the activities of daily living and socialization (Dijkerman et al., 2004). It is well-documented that upper and lower extremity weakness in adults with stroke is related to functional disabilities (Pohl et al. 2000), (Kim et al., 2005). In total, about 85 percent of stroke patients show upper limb disorders in the acute stage (Ryerson, 2001), which increases along the way to 55%–75% after 3–6 months post-stroke (Olsen TS, 1990). A stroke involving the anterior cerebral artery (ACA) will affect the precentral gyrus, presenting with contralateral leg weakness with upper motor signs (Schneider and Gauiter, 1994). The movement limitations from the permanently affected upper limb for performing activities of daily living (Akbari et al., 2011) do not make it easy for post-stroke patients to live normal lives (Kim et al., 2014), emphasizing the importance of improvement of the upper limb functions in the rehabilitation treatment regimens (Paik and Kim, 2010).

Grip strength has been suggested as an important variable to measure after strokes. Boissy et al. (1999) investigated grip strength in 15 persons with chronic stroke and demonstrated that the strength in the more affected hand was significantly associated with the degree of disability of the upper extremity. They also showed that persons with equal grip strength in the more affected hand had almost normal upper extremity function. Moreover, in longitudinal studies, grip strength has been shown to predict motor function in the upper extremity from a short-term and long-term perspective (Heller et al., 1987; Sunderland et al., 1989).

Our findings on grip strength after centrifugation showed a significant improvement in both the normal and paretic sides, but regarding the normal side (R), the grip max. strength% (MS%) and the max. strength (MSKg) had statistically significant improvement with protocol B (a more intense protocol) compared to protocol A, with no significant differences when exercise was added to the centrifugation (Tables 4, 5). This might be due to mild-intensity exercise. But for the paretic side (L), protocol A combined with exercise was more effective for grip strength with less max. strength deficit. Grip exercise by the non-paretic hand was found to be effective in increasing the venous flow volume in the paretic hand, in accordance with the literature (Hayashi and Abe, 2020).

**TABLE 5:** Comparison of protocols for the grip variables.

Variables	Protocol a	Protocol B	+Exercise	Hemiparietic L. side (mean ± sd)	Normal R. side (mean ± sd)
Grip max. strength% (MS%)	✓	—	—	13.7 ± 3.22	86.3 ± 3.22
	✓	—	✓	16.8 ± 2.94	82.5 ± 3.24
	—	✓	—	11.1 ± .866	88.9 ± .977
	—	✓	✓	14.5 ± 2.31	85.5 ± 2.31
Max. strength (MSKg)	✓	—	—	3.11 ± 1.13	19.0 ± 3.75
	✓	—	✓	4.4 ± .826	21.2 ± 1.93
	—	✓	—	3.06 ± .22	24.2 ± .973
	—	✓	✓	3.77 ± .756	22.1 ± 1.95
Maximum strength deficit percentage (MSD%)	✓	—	—	84.0 ± 4.44	
	✓	—	✓	79.4 ± 4.52	
	—	✓	—	87.4 ± 1.17	
	—	✓	✓	82.9 ± 3.10	

For the normal side (R), the grip max. strength% (MS%) and the max. strength (MSKg) of protocol B was more effective than that of protocol A (dark color). For the paretic side (L), protocol A combined with exercise was more effective (dark color). Less max. strength deficit appeared with protocol A combined with exercise. The tick stands for statistically significant improvement, and the negative sign stands for no statistically significant improvement.

The decrease of muscle force in the paretic upper limb may be due to neural factors that affect motor control and also due to inactivity, which contributes to changes in muscle fibers and atrophy (Patten et al., 2004). For patients with adequate motor control, both resistive and aerobic exercises performed 3–4 times a week for a period of 3 months have been found to improve strength and functional activities. Trials revealed that even when the exercise is initiated years after a stroke, it shows gains with exercise (Saunders et al., 2004).

Recognizing the clinical significance of post-stroke weakness in this population leads to the support of the concept that strength training could be a simple approach for improving motor function and reducing disability, even with the presence of spasticity in the affected limb (Hsu et al., 2002; Clark et al., 2006; Tripp and Harris, 1991). According to Garcia-Cabo a patient with muscle weakness after a stroke can improve strength without negative effects, such as worsening spasticity or hypertonia (García-Cabo and Lopez-Cancio, 2020).

Impaired balance is one of the major problems following a stroke. Moreover, balance impairment has consistently been identified as a risk factor for falls in people with stroke (Batchelor et al., 2012). Balance relies on the complex interaction of neural and musculoskeletal systems, e.g., good balance requires the ability of the muscles, particularly of the lower extremities, to produce adequate force at the appropriate time (Pollock et al., 2000; Carr and Shepherd, 2010b). The balance and stance variables in the present study were significantly improved with centrifugation, depending upon the type of protocol and the side measured. The weight distribution was improved in the normal side with both protocols A and B with no significant differences when combined with exercise (maybe the normal side needed a higher exercise load) but for the paretic side, exercise enhanced the effectiveness when combined with protocol A. The force plate parameters indicated greater levels of significance in the anterior/posterior direction compared to medial/lateral components. For the total range, the longitudinal axis was mainly improved with protocol A combined with exercise. The total CoP displacement showed reduced (smaller variance indicative of improvement in balance abilities) by protocol A (larger COP deflections are associated with less stable balance and in a next step with aging and disease). The average velocity was improved by protocol B combined with exercise. Since balance is important for functional activities such as sitting, sit-to-stand, and walking (Carr and Shepherd, 2010a), decrements in balance performance potentially impair activities of daily living.

To objectively determine the patient’s ability or inability to safely balance during a series of predetermined tasks, the Berg Balance Scale test was performed with significant improvement of the score in the rehabilitation period. This balance score is associated with quality of life (Schmid et al., 2013) and is a sensitive (80%) and specific (78%) tool for identifying individuals at risk of falling following a stroke (Maeda et al., 2009). Additionally, the 6MWT was significantly improved from 3 m with a walking aid (cane) to 100 m.

Intermittent artificial gravity *via* centrifugation, a multi-system or integrated countermeasure (Clement and Pavy-Le Traon, 2004), has shown to be beneficial in ambulatory (Evans et al., 2004) and bed-rested subjects (Stenger et al., 2012). Our findings on cardiovascular parameters are similar to previous research studies, which have shown a positive effect of centrifugation with improvement of cardiovascular responses to orthostatic stress (Isasi et al., 2022; Goswami et al., 2015), especially if centrifugation is combined with exercise (Greenleaf et al., 1997; Iwase et al., 2002; Iwase, 2005). In the present study, the improvement of cardiovascular markers over time (Figures 7, 8) showed the effectiveness of the training device in improving the patient’s physical condition.

The main mechanism of the effect of AG on the human body in the head-to-toe direction is blood pooling in the veins of the lower part of the body, which may cause a sudden decrease in blood pressure, initiating a stress condition that the cardiovascular system needs to overcome in order to assure blood circulation to all parts of the body. Higher levels of AG increase the stress upon the cardiovascular system and leads to more intense cardiovascular reflex responses in order to maintain blood pressure homeostasis. This leads to inhibition of parasympathetic activity by the autonomic nervous system and increase of sympathetic stimulation triggered by the primary receptors (the arterial baroreceptors in the carotid sinus and the aortic arch areas) for short-term control of the cardiovascular system. We noticed that these responses were more intense when cardiovascular demand increased further due to the combination of SAHC with exercise, and specifically, CO increased significantly to confront the new metabolic demands imposed by the exercise activity (Guyton and Hall, 1996).

Furthermore, muscle activation increased mean systemic pressure due to venous return, and autonomic stimulation increased heart rate and heart contractility. Generally, we noticed a statistically significant effect in all cardiovascular variables between any of our exercise combinations. An increase in heart rate and contractility of the heart acts to maintain the appropriate amount of blood flow to all the organs, in particular the brain. Animal studies have confirmed increased blood flow to the cerebral cortex and brain stem with centrifugation and attribute the changes observed to the central and peripheral vascular systems (Adams et al., 2001). It is likely that the same changes observed in the central and peripheral vascular system, would also take place in the central nervous system vascular interstitial fluid. Rampello et al. (2007) demonstrated that cardiorespiratory training may be better than neurorehabilitation.

Caliandro et al. (2017) demonstrated that stroke may cause changes in small-world characteristics of the cortical network organization as measured by graph theory methodology applied on cortical sources of EEG data. In the present study, significant improvement was revealed in precentral and postcentral gyrus areas after treatment with SAHC alone of combined to exercise, as was identified by graph theory on the cluster coefficient, betweenness centrality metric, and node degree in pre and postcentral gyrus. The cluster coefficient and betweenness centrality metric were affected by both protocols alone, and it seems that exercise added marginally to the final effect, while the node degree presented a significant difference only when SAHC was combined with exercise. The precentral gyrus, located in the frontal lobe and on both sides of the brain, is referred to as the primary motor cortex or as the motor strip. While the planning of movements occurs in the frontal lobe, all the information is processed to the motor strip prior to performance. The left side of the motor strip controls all movement on the right side of body, while the right side controls all movement on the left side. The proper functioning of the motor strip also needs proper development of the muscular, skeletal, and nervous systems to enable a person to move.

A lesion in the right hemisphere, as in the present case, leads to difficulty to shift the body weight between the legs, poor body vertical orientation, body sway, and balance control (Fernandes et al., 2018; Coelho et al., 2019). In the present case, in both right pre- and post-central gyrus, cluster coefficient betweeness and node degree improved significantly after treatment with higher levels when SAHC was combined with exercise, indicating neuron recruitment and neuroplasticity. The effectiveness of the treatment and recovery of neurotransmission after a stroke may be an indication of neuroplasticity (Binkofski and Seitz, 2004; Carmichael et al., 2004). The underlying mechanism may be by enhancing the recruitment of the neurons in both hemispheres of the brain that contribute to performance, increase communication among neurons, and strengthen synaptic connections, especially in the affected side of the brain. This may lead to the improvement of cognitive, language, and motor skills by means of the cerebral processes involved in ordinary learning.

### 4.1 Mechanism of SAHC alone as a whole-body exercise-mimicking device

Exercise therapy helps stroke patients relearn lost movement patterns. Upper limb functions, muscle strength, balance, and walking ability can be improved. Lower limb training can reduce spasticity, improve strength and endurance, and assist coordination (Kamps and Schule, 2005). Functional neuroimaging studies have shown the evolution of cerebral activity within both hemispheres as patients’ skills improve with training and experience (Baron et al., 2004). Centrifugation consists of uniformity and repetitiveness of the exercises at a certain constant pace, which may play an important role in stroke rehabilitation by stimulating the brain’s ability to reorganize itself (Plautz et al., 2000). This allows healthy areas of the brain to take over the function of the affected areas. The controlled motion of the uniform centripetal acceleration of the SAHC, a passive movement therapy device, allows exactly this kind of repetitive movement training.

Furthermore, CNS plasticity may be enhanced by muscle activity according to the CNS plasticity model, in part, through the affective signals sent by muscle and joint receptors triggered by movement. The most important is the quality of the movement, which needs to be close to the normal performance as possible and sufficiently repeated. In the case of stroke patients, they may have movements by muscle contractions (Quaney et al., 2009), but they are not normal or sometimes do not exist. There is a need to introduce effective interventions for those stroke patients who are moderately or severely involved. Considering the evidence of CNS plasticity and associated principles of motor learning, we introduced SAHC to mitigate the detrimental effects of bed rest and we mainly focus on the effects of artificial gravity as a whole body physical exercise within a multi-factorial framework in which a variety of lifestyle factors, or pharmacological treatments, are measured and manipulated, taking into account the genetic predispositions for dementia, disease, or cognitive dysfunction (variation in genes, such as the BDNF gene), the efficacy of aerobic exercise in relation to symptom severity, duration of illness, comorbidity of diseases, the brain areas, and molecular factors most affected in the disease, as well as possible interactions with pharmaceutical treatments, and finally, a more refined task manipulations in the context of SAHC intervention will enable a detailed characterization of the cognitive processes that are most affected by artificial gravity and whether such cognitive and neural benefits are transferred to everyday functions.

Stroke patients can train easily and effectively with this device independently of their degree of disability on an individualized training program, lying on a bed with or without additional aerobic or resistive exercise.

The present study validated individualized AG protocols that couple the countermeasure needs of skeletal muscle, bone, and cardiovascular and neurological systems. It also proposed a multi-modal evaluation framework for validating the beneficial role of artificial gravity in the prevention of the physiological deconditioning that normally occurs in microgravity and other bed rest and reduced mobility conditions. Therefore, the SAHC training could be regarded as a new gravity-focused therapeutic approach for rehabilitation interventions.

The present case study shows promising results regarding the utility of the artificial gravity training as a stroke rehabilitation approach. However, forthcoming randomized controlled trials are needed to validate the statistical significance of the results on a large population cohort. The inclusion of either a control group or the comparison with current state of the art approaches is expected to provide concrete scientific evidence. We acknowledge this major methodological limitation of the current approach and aim to address it within the activities of the H2020-funded project, VITALISE No.101007990—https://vitalise-project.eu/ (Bernaerts et al., 2022).

Brain plasticity after stroke refers to the regeneration of brain neuronal structures and/or reorganization of the function of neurons. Not only can CNS structure and function change in response to injury but also the changes may be modified by “activity”. For gait training or upper limb functional training for stroke survivors, the “activity” is motor behavior, including coordination and strengthening exercises and functional training that comprise motor learning. The ultimate goal of rehabilitation is to restore function so that a satisfying quality of life can be experienced.

The present case study proposes a novel infrastructure based on the SAHC to investigate the hypothesis that artificial gravity ameliorates the degree of disability resulting from a variety of circumstances from mobility to cognitive integrity and function.

## Data Availability

The raw data supporting the conclusion of this article will be made available by the authors, without undue reservation.
