# Supplementary material for: Rehabilitation assisted by Space technology—A SAHC approach in immobilized patients—A case of stroke
Source: Front Physiol. 2023 Jan 18;13:1024389. doi: 10.3389/fphys.2022.1024389 (PMC9890276; doi:10.3389/fphys.2022.1024389)
Supplement: Supplementary file 1 [file DataSheet1.docx]

The Tukey post-hoc comparisons for each cardiovascular marker are the following:

### Cardiac Output

There was a statistically significant main effect of condition when the intervention was Protocol B with exercise: F (1,388) =10.3, p=0.001. There was also a statistically significant main effect of protocol, when there was exercise and for Condition 2 (1.5g): F (1,388) = 4.94, p=0.027.

### Cardiac Index

There was a statistically significant main effect of condition when the intervention was Protocol B with exercise: F (1,388) =10.7, p=0.001. There were also statistically significant main effects of protocol, when there was a) exercise and Condition 1 (Lying): F(1,388) = 11.8, p=0.000643, b) ) exercise and Condition 2 (1.5g): F(1,388) = 16.4, p=0.000627, c) ) exercise and Condition 3 (1.7g – 1^st^ session): F(1,388) = 3.9, p=0.049, d) no exercise and Condition 6 (1.7g – 4^th^ session): F(1,388) = 4.59, p=0.033, e) exercise and Condition 6 (1.7g – 4^th^ session): F(1,388) = 5.60, p=0.018.

### Stroke Volume

There were statistically significant main effects of Exercise when: a) Protocol A – Condition 1 (Lying): F (1,388) = 6.92, p=0.009, b) Protocol A – Condition 5 (1.7g – 3^rd^ session): F (1,388)=4.14, p=0.043, c) Protocol A – Condition 6 (1.7g – 4^th^ session): F (1,388)=10.80, p=0.001, d) Protocol B – Condition 6 (1.7g – 4^th^ session): F (1,388)=10.20, p=0.001.

### Stroke Volume Index

There were statistically significant main effects of Exercise when: a) Protocol A – Condition 1 (Lying): F(1,388) = 6.22, p=0.013, b) Protocol A – Condition 5 (1.7g – 3^rd^ session): F(1,388)=4.58, p=0.033, c) Protocol A – Condition 6 (1.7g – 4^th^ session): F(1,388)=11.00, p=0.00097, d) Protocol B – Condition 6 (1.7g – 4^th^ session): F(1,388)=10.10, p=0.002.

### Pulse Rate

Tukey post-hoc comparisons identified a marginally significant main effect of condition for Protocol A with no exercise [F(1,388)=3.16, p=0.076] and statistically significant effects for a) Protocol A with exercise [F(1,388)=10.7, p=0.001], b) Protocol B with no exercise [F(1,388)=22.8, p<0.0001] and c) Protocol B with exercise [F(1,388)=10.6, p=0.001].

When there was no exercise, there were statistically significant protocol effects for a) Condition 1 (Lying) [F (1,388) =6.94, p=0.009], b) Condition 5 (1.7g – 3^rd^ session) [F (1,388) =11.9, p=0.0006] and c) Condition 6 (1.7g – 4^th^ session) [F (1,388) =9.25, p=0.003].

When there was exercise, there were statistically significant protocol effects for a) Condition 1 (Lying) [F (1,388) =9.30, p=0.002], b) Condition 2 (1.5g) [F (1,388) =13.2, p=0.0003] and c) Condition 6 (1.7g – 4^th^ session) [F (1,388) =5.08, p=0.025].

There were statistically significant exercise effects for a) Condition 2 (1.5g) and Protocol A [F (1,388) =4.59, p=0.033], b) Condition 6 (1.7g – 4^th^ session) & Protocol A [F(1,388)=6.30, p=0.012], and c) Condition 6 (1.7g – 4^th^ session) & Protocol B [F(1,388)=7.35, p=0.007].

Marginal significance was reached for a) Condition 2 (1.5g) and Protocol B [F (1,388) =3.54, p=0.061] and b) Condition 5 (1.7g – 3^rd^ session) and Protocol B [F (1,388)=3.3, p=0.07].

### Systolic Pressure

Tukey post-hoc comparisons showed statistically significant Condition effects for a) Protocol A with no exercise [F (1,388) =11.7, p=0.0007], b) Protocol B with no exercise [F (1,388) =7.17, p=0.008], c) Protocol A with exercise [F (1,388) =25.0, p<0.0001] and d) Protocol B with exercise [F (1,388) =38.7, p<0.0001].

There were also statistically significant Exercise effects for a) Condition 2 (1.5g) and Protocol B [F (1,388) =7.6, p=0.006], b) Condition 4 (1.7g – 2^nd^ session) and Protocol A [F (1,388) =7.29, p=0.007], and c) Condition 6 (1.7g – 4^th^ session) and Protocol B [F (1,388) =8.32, p=0.004]. There was also a marginally significant effect for Condition 3 (1.7g – 1^st^ session) and Protocol A [F (1,388) =3.69, p=0.056].

Statistically significant Protocol effects were reported for a) Condition 2 (1.5g) with exercise [F (1,388) =6.06, p=0.014], b) Condition 3 (1.7g – 1^st^ session) with exercise [F (1,388) =7.31 p=0.0], c) Condition 4 (1.7g – 2^nd^ session) with exercise [F (1,388) =5.45, p=0.02].

There were also marginally significant differences for a) Condition 1 (Lying) with exercise [F (1,388) =3.68, p=0.0] and b) Condition 6 (1.7g – 4^th^ session) with no exercise [F (1,388) =3.63, p=0.057].

### Mean Arterial Pressure

Tukey post-hoc comparisons showed statistically significant Condition effects for a) Protocol A with no exercise [F (1,388) =13.5, p=0.0003], b) Protocol B with no exercise [F (1,388) =5.32, p=0.022], c) Protocol A with exercise [F (1,388) =21.0, p<0.0001] and d) Protocol B with exercise [F (1,388) =25.3, p<0.0001].

There was a marginally significant Protocol effect for Condition 4 (1.7g – 2^nd^ session) with exercise [F (1,388) =3.46, p=0.063] and a statistically significant effect for Condition 4 (1.7g – 2^nd^ session) with no exercise [F (1,388) =5.04, p=0.025].

Finally, there were statistically significant Exercise effects for a) Condition 2 (1.5g) with Protocol B [F (1,388) =4.55, p=0.033], b) Condition 4 (1.7g – 2^nd^ session) with Protocol A [F (1,388) =6.84, p=0.009] and c) Condition 6 (1.7g – 4^th^ session) with Protocol B [F(1,388)=4.47, p=0.035].
